# Regulation of Long Noncoding RNAs Responsive to Phytoplasma Infection in *Paulownia tomentosa*

**DOI:** 10.1155/2018/3174352

**Published:** 2018-02-21

**Authors:** Guoqiang Fan, Yabing Cao, Zhe Wang

**Affiliations:** ^1^Institute of Paulownia, Henan Agricultural University, Zhengzhou, Henan 450002, China; ^2^College of Forestry, Henan Agricultural University, Zhengzhou, Henan 450002, China

## Abstract

Paulownia witches' broom caused by phytoplasma infection affects the production of Paulownia trees worldwide. Emerging evidence showed that long noncoding RNAs (lncRNA) play a protagonist role in regulating the expression of genes in plants. So far, the identification of lncRNAs has been limited to a few model plant species, and their roles in mediating responses to *Paulownia tomentosa* that free of phytoplasma infection are yet to be characterized. Here, whole-genome identification of lncRNAs, based on strand-specific RNA sequencing, from four *Paulownia tomentosa* samples, was performed and identified 3689 lncRNAs. These lncRNAs showed low conservation among plant species and some of them were miRNA precursors. Further analysis revealed that the 112 identified lncRNAs were related to phytoplasma infection. We predicted the target genes of these phytoplasma-responsive lncRNAs, and our analysis showed that 51 of the predicted target genes were alternatively spliced. Moreover, we found the expression of the lncRNAs plays vital roles in regulating the genes involved in the reactive oxygen species induced hypersensitive response and effector-triggered immunity in phytoplasma-infected Paulownia. This study indicated that diverse sets of lncRNAs were responsive to Paulownia witches' broom, and the results will provide a starting point to understand the functions and regulatory mechanisms of Paulownia lncRNAs in the future.

## 1. Introduction

Plant witches' broom is an epidemic disease, which is caused by specialized obligate bacteria (i.e., phytoplasma) and spread by insect vectors [1]. To date, over 1000 plant species have been found to be affected by phytoplasma worldwide, including Paulownia [2], mulberry [3], Chinese jujube [4], grape [5], and lime [6]. Phytoplasma-infected plants often undergo a series of physiological and biochemical changes, that induce drastic malformations, such as short internodes, dwarfism, proliferation of axillary buds (witches' broom), yellowing of leaves, flower sterility, and even dieback of plants [7], which have had a devastating effect on agriculture, forestry, and horticultural crop production.

Paulownia is a fast-growing deciduous hardwood tree species native to China, which plays a leading role in improving the ecological environment [8]. However, phytoplasma, which belongs to the aster yellows group “*Candidatus* Phytoplasma asteris” (16SrI-D), invasion in Paulownia often leads to slow growth and even death of trees, which results in enormous economic losses [9], and the genome sequencing of this phytoplasma has not yet been completed. Since the disease was first reported by Doi in 1967, lots of research have been carried out on the diagnosis [10], preservation [11], distribution and concentration changes with seasonal variation [12], and the molecular mechanisms of Paulownia witches' broom (PaWB) infection; other studies on phytoplasma genome and virulence factors have also been carried out [13, 14]. However, to date, no clear mechanisms behind its molecular regulation has been found, mostly due to the complexity of PaWB phytoplasma itself and the limitations of current technical methods. With the rapid development of high-throughput “omics” technologies, transcriptome, microRNA (miRNA), proteome, and metabolome data have become available and have been used to analyze variations in Paulownia after phytoplasma infection; as a result, a series of genes, miRNAs, proteins, and metabolites that are potentially related to the occurrence of PaWB have been identified [2, 15–23]. Despite this, the molecular mechanism of PaWB is still poorly understood. Because, though large numbers of genes, miRNAs, proteins, and metabolites have been reported, the correlation among them is low. Recent studies have shown that changes in the expression levels of long noncoding RNAs (lncRNAs) were closely related to plant growth and development and to biotic and abiotic stress responses in numerous organisms [24].

lncRNAs are defined as having more than 200 nucleotides and little protein-coding potential [25]. Usually, lncRNAs have time- or tissue-specific expression patterns and execute their functions in four main ways, that is, as signals, decoys, guides, or scaffolds [26]. To date, several plant species, including *Arabidopsis thaliana* [27], *Zea mays* [28], *Triticum aestivum* [29], *Oryza sativa* [30], *Populus tomentosa* [31], *Capsicum annuum* [32], *Selaginella moellendorffii* [33], and *Brassica napus* [34], have conducted to understand roles and mechanism of lncRNAs. In *Arabidopsis*, two lncRNAs, *COOLAIR* and *COLDAIR* can regulate flowering time through promoter interference and histone modification, respectively [35], and lncRNAs induced by phosphate starvation response 1 (PHR1) were also identified as the target gene of miRNA399 [36]. Furthermore, lncRNAs involved in responses to biotic stresses have also been found in plants. For instance, In *T. aestivum* infected with the fungus *Blumeria graminis* f. sp. *tritici*, two lncRNAs, *TaS1* and *TaS2*, were found to play important roles in the response to pathogen infection [37]. Similarly, lncRNAs as target mimics for miRNAs in tomatoes infected with tomato yellow leaf curl virus have also been identified [38]. All these results support the idea that lncRNAs may play significant roles in the regulation of plant growth and development, differentiation, and stress responses. However, the roles of lncRNAs in phytoplasma-infected woody plants are still unknown.

In the present study, we used high-throughput strand-specific RNA sequencing (RNA-seq) to elucidate the expression profiles of lncRNAs in healthy (PT), phytoplasma-infected (PTI), healthy 60 mg·L^−1^ MMS-treated (PT-MMS), and phytoplasma-infected 60 mg·L^−1^ MMS-treated (PTI-MMS) *Paulownia tomentosa* cuttings. We identified 112 lncRNAs that were differentially expressed in response to phytoplasma infection. This study will increase our understanding of phytoplasma-responsive lncRNAs and lay a solid foundation for further clarifying the functions of these phytoplasma-responsive lncRNAs.

## 2. Materials and Methods

### 2.1. Plant Material and Methyl Methanesulfonate (MMS) Treatment

All of the tissue-cultured cuttings used in this study were obtained from the Institute of Paulownia, Henan Agricultural University, Zhengzhou, Henan Province, China. Shoot tips from healthy *P. tomentosa* cuttings (PT) and PaWB-infected cuttings (PTI) were cultured for 30 days before being clipped. The uniform shoot tips from the PTs and PTIs were transferred into 1/2 MS culture medium containing 25 mg·L^−1^ sucrose and 8 mg·L^−1^ agar (Sangon, Shanghai, China) with 0 or 60 mg·L^−1^ MMS, respectively. PTs without MMS were used as the control. At least 120 samples, including 30 PTs, 30 PTIs, 30 PT-MMS, and 30 PTI-MMS, were prepared. The cultivation procedure was performed as described in Fan et al. [17]. Shoot tips about 1.5 cm in length were collected from these 30 cuttings and were mixed to form one biological replicate, then immediately frozen and ground in liquid nitrogen and stored at −80°C for RNA and DNA extraction.

### 2.2. PaWB Phytoplasma Detection

Total DNA was isolated from the PT, PTI, PT-MMS, and PTI-MMS using the cetyl trimethyl ammonium bromide (CTAB) (Beijing Chemical Co., Beijing, China) method as described by Zhang et al. [39], respectively. PaWB phytoplasma was detected by nested PCR. PCR amplification and agarose gel electrophoresis were performed as described by Fan et al. [40].

### 2.3. Strand-Specific RNA Library Construction and RNA Sequencing

Total RNAs were extracted from the PT, PTI, PT-MMS, and PTI-MMS using TRIzol reagent (Invitrogen, Carlsbad, CA, USA) according to the manufacturer's instruction, respectively. Total RNA quality and quantity were assessed using a NanoDrop ND-1000 spectrophotometer (Thermo Scientific, Waltham, USA) and Agilent Bioanalyzer 2100 (Agilent Technologies, Palo Alto, CA, USA) according to the manufacturer's instructions. RNA samples with an OD260/280 nm ratio of 2.0 to 2.1 were used for the later analysis. A Ribo-Zero™ Magnetic kit was used to remove the rRNA. The four RNA samples (PT, PTI, PT-MMS, and PTI-MMS) were used to construct strand-specific RNA-seq libraries according to the TruSeq RNA Sample Preparation Guide. The libraries were sequenced on an Illumina HiSeq™ 2000 platform at Beijing Genomics Institute (Shenzhen, China).

### 2.4. Transcript Assembling and lncRNA Prediction

High-quality pair-end RNA-seq reads were obtained by removing low-quality reads with more than 50% of the bases with a Q ≤ 10, discarding reads with adaptor sequences, and reads with more than 10% “N” bases. To remove rRNA reads, the high-quality reads were aligned to sequences in the SILVA ribosomal RNA (rRNA) gene database (http://www.arb-silva.de/) using SOAP2, with a maximum of 5 mismatches allowed for each alignment to each read. All the remaining clean reads were assembled using Trinity. Because the complete *P. tomentosa* genome was not available at the time of this study, the clean reads were mapped to the *P. fortunei* reference genome (http://paulownia.genomics.cn) using TopHat2 with no more than 2 mismatches. The mapped reads were used to assemble the transcripts in each sample using Cufflinks 2.0 program [41]. Low-quality assemblies with transcript length< 200 bp were discarded. Novel transcripts were discovered by filtering out the known transcripts that mapped to the annotated reference gene sequence. Finally, the coding potential calculator was used to screen for putative lncRNAs (with coding potential calculator scores < 0) among the novel transcripts. Based on their location in the reference genome, these lncRNAs were categorized into five classes: intergenic (lncRNA located in intergenic region), intronic (lncRNA derived wholly from intron), sense, antisense (lncRNA overlapping one or more exons of another transcript on the same or opposite strand), and bidirectional (the expression of lncRNA and a neighboring coding transcript on the opposite strand).

### 2.5. Identification of Conserved lncRNAs

We set two different criteria to discover conserved lncRNAs in the lncRNA database CANTATAdb (http://yeti.amu.edu.pl/CANTATA/): (i) all the lncRNA sequences identified in this study were aligned against the lncRNA sequences in CANTATAdb using BLASTN with a cutoff *E* value < 1*E* − 5 and (ii) lncRNA sequence identity to lncRNA sequences in other plant genomes was >20% [42]. In addition, lncRNAs that may act as the miRNAs precursors were predicted by aligning the lncRNA sequences to the miRNA sequences of Paulownia using BLASTN. PsRNATarget (http://plantgrn.noble.org/psRNATarget/) was used to predicted lncRNAs as targets of miRNAs with *E* value ≤ 3. Target mimics were predicted according to the rules described by Wu et al. [43].

### 2.6. Prediction of the Potential Target Genes of PaWB Responsive lncRNAs

In this study, two independent algorithms, cis- or trans-acting, were used to predict potential targets of the PaWB responsive lncRNAs in the PT, PTI, PT-MMS, and PTI-MMS cuttings according to their regulatory mechanism. The first algorithm predicted potential target genes of cisacting lncRNAs that were physically located within 10 kb upstream or 20 kb downstream of lncRNAs using a genome browser. The second algorithm predicted potential target genes of trans-acting lncRNAs based on the lncRNA-mRNA (target) sequence complementary and predicted lncRNA-mRNA duplex energy. First, BLASTN searches were performed to detect potential target mRNA sequences complementary to the lncRNA sequences with identity > 95% and *E* value < 1*E* − 5. Then we used the RNAplex software to calculate the complementary energy between the lncRNAs and their potential transregulated target genes with RNAplex − *E* ^−30^.

### 2.7. Comparison of lncRNA Expression among the Different Samples

Several pairwise comparisons were carried out among the four samples to search for candidate lncRNAs related to PaWB formation (Figure S1). (i) Differentially expressed lncRNAs selected from the PTI-MMS versus PTI comparison may be related to the influence of the methylating agent MMS and PaWB; (ii) differentially expressed lncRNAs in PT-MMS versus PT comparison are likely to be related to the influence of MMS; (iii) differences between comparisons 1 and 2 may exclude differentially expressed lncRNAs related to the influence of MMS; (iv) differentially expressed lncRNAs in the PTI versus PT comparison are likely to be involved in PaWB; (v) and the common lncRNAs between comparisons 3 and 4 may be directly related to PaWB.

### 2.8. Screening of Differentially Expressed lncRNAs Related to PaWB Disease and Functional Prediction

The calculation of lncRNA expression levels from the four different samples was normalized to the FPKM value. The false discovery rate (FDR), a commonly used statistical method in multiple tests, was used to determine the threshold of the *P* value [44]. Differentially expressed lncRNAs were judged with an absolute value of log2 ratio > 1 or <−1 and a threshold FDR < 0.001. To better understand the biological processes regulated by these differentially expressed lncRNAs, the sequences of the potential target genes of these lncRNAs were aligned to the Nr, Swiss-Prot, KEGG, and COG databases using BLASTX (*E* value < 1.0*E* − 5) and searched against the InterPro database using the InterProScan software package.

### 2.9. Quantitative Real-Time Polymerase Chain Reaction (qRT-PCR) Analysis

Total RNA (1 *μ*g) obtained from leaves of four samples were reverse transcribed into cDNA for validating the expression of lncRNAs, miRNAs predicted to target lncRNAs, and potential target genes of lncRNAs by real-time quantitative PCR (qRT-PCR). qRT-PCR analyses were performed on a StepOne Plus real-time PCR system (Life Technologies, Burlington, ON, Canada) using FASTSYBR green mix from Kappa Biosystem (D Mark, Toronto, ON, Canada). All the primers were designed using Primer Express 5.0 (Applied Biosystems). The primer sequences used in the qRT-PCR analyses are provided in Table S1. All the amplifications were carried out in triplicate, with the standard reaction program (94°C for 3 min, followed by 40 cycles of 94°C for 10 s, and 58°C for 30 s, finally, 72°C for 30 s). The specificity of the amplified fragments are checked using the generated melting curve. The generated real-time data were analyzed using the Opticon Monitor Analysis Software 3.1 tool and standardized to the levels of 18S rRNA (lncRNA and there corresponding target genes) and U6 (miRNA) using the 2^−ΔΔCt^ method [45].

## 3. Results

### 3.1. Detection of Phytoplasma in *P. tomentosa* Cuttings Showing Symptoms of Witches' Broom

The phytoplasma-infected *P. tomentosa* (PTI) cuttings showed drastic malformations, including short internodes, proliferation of axillary buds, and yellowing leaves. When treated with 60 mg·L^−1^ MMS, the PTI-MMS cuttings regained a healthy morphology, while the healthy cuttings treated with 60 mg·L^−1^ MMS (PT-MMS) showed no obvious changes (Figure S2). Fragment of the 16S rDNA sequence from the PaWB phytoplasma genome was detected in the PTI samples but not in the PT, PT-MMS, or PTI-MMS samples (Figure S3). These results suggest that phytoplasmas have disappeared after the 60 mg·L^−1^ MMS treatment.

### 3.2. Genome-Wide Identification and Characterization of lncRNAs in Phytoplasma-Infected *P. tomentosa*

**Figure 1:**
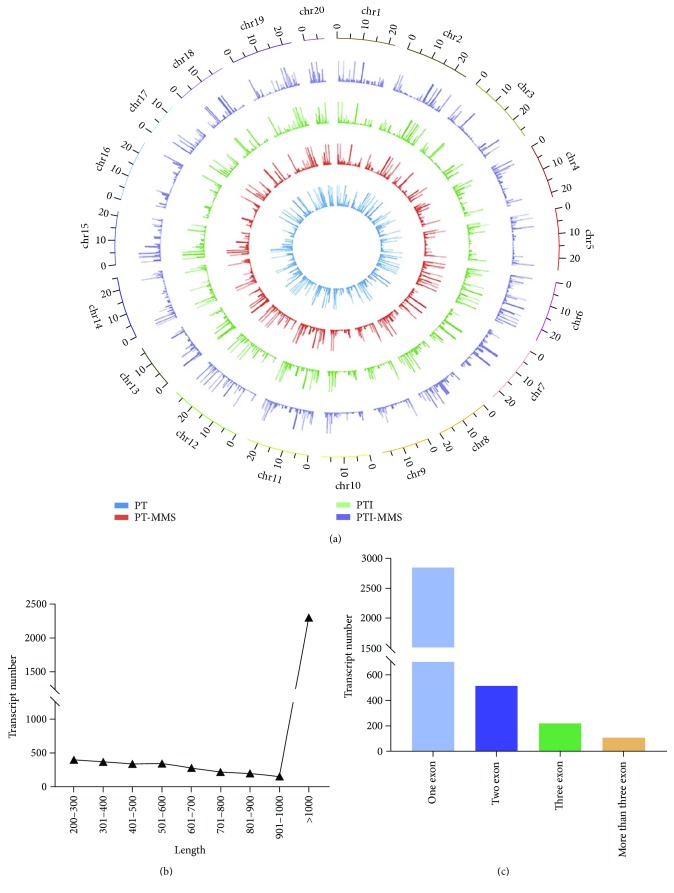
Features of lncRNAs in *P. tomentosa*. (a) Distribution of lncRNAs along each chromosome. (b) Length distribution of 3689 *P. tomentosa* lncRNAs. (c) Number of exons per transcript for all lncRNA transcripts.

To systematically identify *P. tomentosa* lncRNAs responsive to phytoplasma infection, strand-specific RNA-seq was performed for RNA samples from healthy and phytoplasma-infected *P. tomentosa* leaves under 0 or 60 mg·L^−1^ MMS treatment. A total of 208,686,782 pair-end raw reads were obtained from the four libraries. After trimming, 204,943,862 clean reads were obtained. From these reads, 32,283 transcripts were assembled using Cufflink. Among these transcripts, 28,593 were completely aligned against the reference genome of *Paulownia fortunei*, and the remaining transcripts that are not aligned were considered as novel transcripts. With FPKM > 0.5 as the cut-off and using the lncRNAs prediction standard, 3689 lncRNAs were identified in the four libraries (Table S2), these included 45 bidirectional lncRNA, 563 antisense lncRNAs, 3012 intergenic lncRNAs, and 69 sense lncRNAs. Thus, the intergenic lncRNAs made up 81.6% of the total *P. tomentosa* lncRNAs, which is consistent with the previous study [34]. The distribution of the lncRNAs in the genome is vital for the genetic manipulation required to adapt to the stress of the phytoplasma infection. By mapping these putative lncRNA sequences to the reference genome sequence, we found that they evenly distributed in each chromosome (Figure 1(a)). The size distribution of the potential lncRNAs identified in this study ranged from 200 to 18,769 bases, with most lncRNAs (68.4%) ranging from 200 to 1000 bases (Figure 1(b)). The average and median lengths of the lncRNAs were 1052 bp and 680 bp, respectively, which is longer than the *Arabidopsis* lncRNA transcripts (median length of 285 bp) but shorter than the lncRNA transcripts of rice (median length of 852 bp) and *Populus tomentosa* (median length of 736 bp) [42]. The average and median lengths of the protein-coding mRNA of Paulownia were 1528 bp and 1245 bp, respectively, which are longer than those of noncoding transcripts. Characterization of the genomic structure of these lncRNAs revealed that 2815 of them had only one exon, 512 had two exons, 219 had three exons, and the remaining lncRNAs had more than three exons (Figure 1(c)).

The conservation of lncRNA is considered to be lower than that of protein-coding genes, so if lncRNAs perform evolutionarily conserved functions, they may be conserved among different species. Thus, to detect conserved lncRNAs, all the lncRNA sequences were searched against the genomes of 10 representative plants (*A. thaliana*, *O. sativa*, *Glycine max, Selaginella*, *Chlamydomonas*, *Physcomitrella*, *Amborella*, *Solanum tuberosum*, *V. vinifera*, and *Z. mays*) using BLAST. The results showed that only a small number of the *P. tomentosa* lncRNAs were conserved across these 10 species (Table 1 and Table S3). The highest number matched to the known lncRNAs in *V. vinifera*, likely because, among these 10 species, Paulownia was most closely related to *V. vinifera* in evolution. To annotate the predicted lncRNAs from an evolutionary point of view, we used INFERNAL to classify them into different noncoding RNA families. Based on their consensus secondary structures, we identified 451 unique sequences belonging to 170 conserved lncRNA families (Table S4); among them, MIR families accounted for most of the conserved lncRNA families, followed by MIR families.

### 3.3. lncRNAs Function as Precursors or Target Mimics of miRNAs

**Figure 2:**
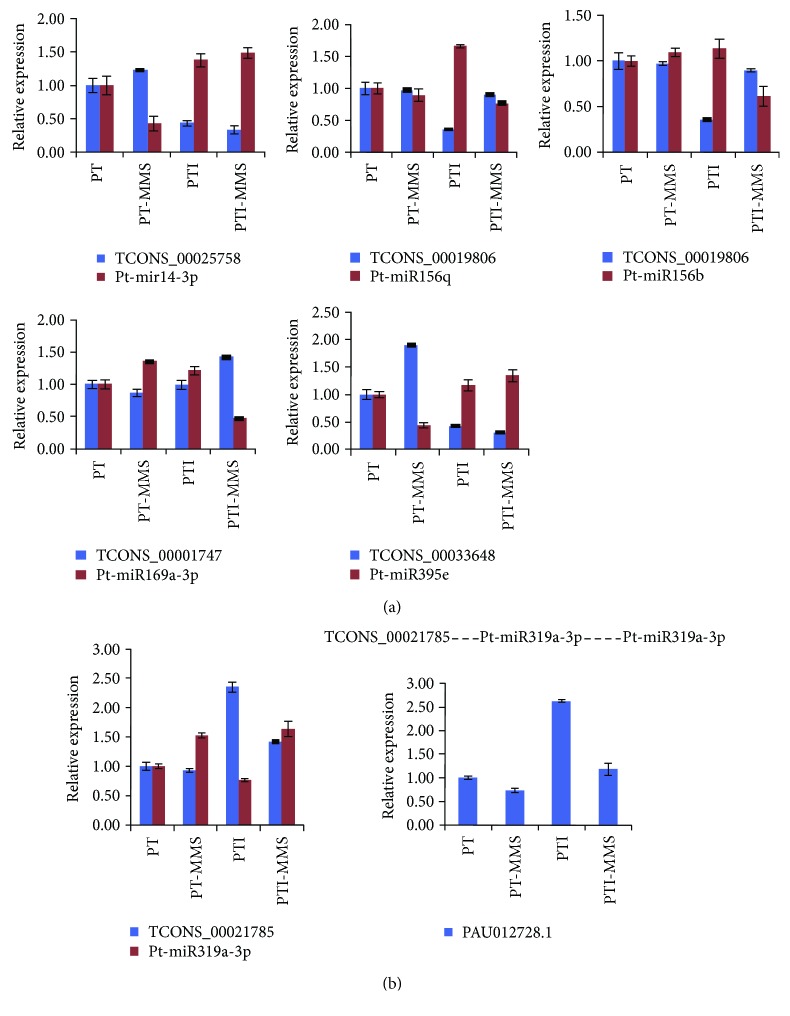
Expression analysis of lncRNAs as potential targets or target mimics of miRNAs. (a) Quantitative RT-PCR analysis of miRNAs and their potential target lncRNAs. (b) Quantitative RT-PCR analysis of TCONS_00021785 and one potential target gene of pt-miR319a-3p (PAU012728.1).

lncRNAs may influence transcriptional, posttranscriptional, and epigenetic gene regulation through miRNAs [46]. We identified lncRNAs that could act as a precursor of known miRNAs in Paulownia. Six lncRNAs were predicted as precursors of 10 known miRNAs belonging to three miRNA families (Table 2). Thus, we speculated that these lncRNAs might function as miRNAs in response to phytoplasma infection. lncRNAs can also regulate gene expression and numerous biological processes by acting as miRNA targets or target mimics in plants [47]. To explore the possibility of lncRNAs as a target of miRNA, all the lncRNAs were aligned against the *P. tomentosa* miRNA sequences using PsRNATarget. Interestingly, 239 out of the 3689 lncRNAs were predicted to be targeted by 228 miRNAs of *Paulownia tomentosa*, including pt-miR156a/b-5p/c-3p/d/e/f-3p/q, pt-miR160a-3p/b/c, and pt-miR167a/b (Table S5). Moreover, in order to investigate the relationship between miRNAs and their target lncRNAs, qRT-PCR was used to measure their expression of four miRNA-lncRNA pairs. As shown in Figure 2(a), a negative relationship between miRNAs and their target lncRNAs was observed, suggesting that miRNAs may lead to the degradation of their corresponding target lncRNAs.

lncRNAs that potentially function as target mimics of miRNAs were predicted according to Wu et al. [43]. We identified 23 lncRNAs that may act as target mimics and may be bound by 33 miRNAs (26 known miRNAs and 7 novel miRNAs) to form 38 miRNA-lncRNA duplexes (Table S6). Among these miRNA target mimics, TCONS_00021785 was identified as the target mimic of the pt-miR319 family. Notably, three known miRNAs (pt-miR6173e-5p, pt-miR156m, and pt-miR156g-5p) and one novel miRNA (pt-mir30-5p) were predicted to be target mimic of two lncRNAs, respectively. That is to say, functions of these miRNAs may be inhibited. To validate it, the expression level of TCONS_00021785 and the potential target genes of pt-miR319a-3p were examined using qRT-PCR. As shown in Figure 2(b), the expression level of TCONS_00021785 was increased and the expression level of target gene PAU012728.1 was also increased in PTI, suggesting that TCONS_00021785 may increase the expression level of PAU012728.1 by interacting with pt-miR319a-3p.

### 3.4. Identification of Phytoplasma-Responsive lncRNAs

**Figure 3:**
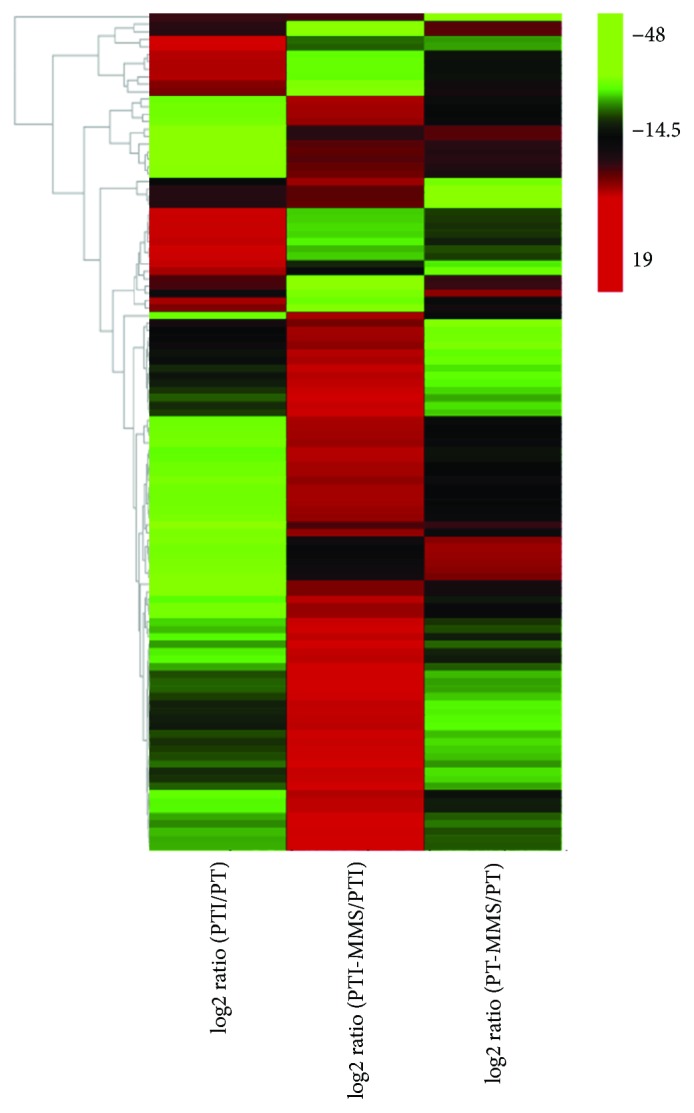
The heatmap of 112 PaWB-related lncRNAs in different comparison.

**Figure 4:**
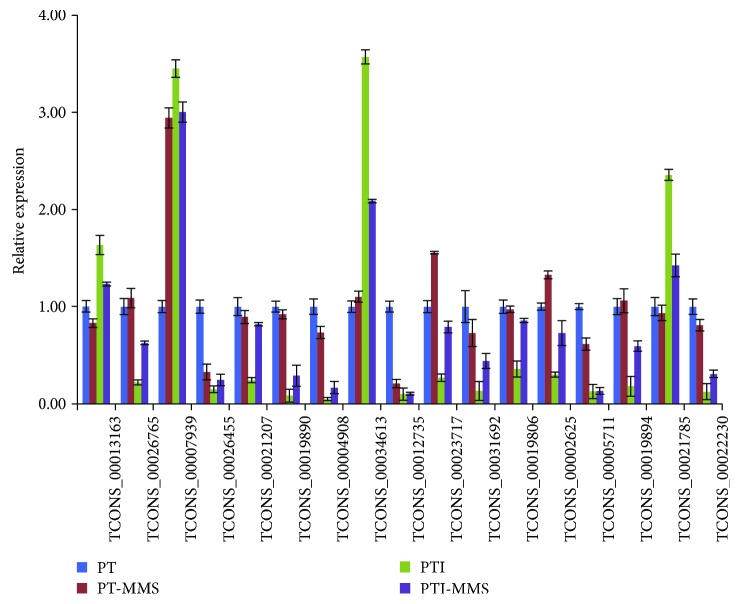
Changes in the relative expression levels of lncRNAs in *P. tomentosa*. Potential target genes of phytoplasma-responsive lncRNAs.

Emerging evidence has demonstrated that lncRNAs are involved in the regulation of the stress regulation response [29, 30]; thus, we analyzed the differentially expressed lncRNAs among the four samples. Differentially expressed lncRNAs were defined as log2 ratio > 1 or <−1, with FDR < 0.001. Accordingly, 728 differentially expressed lncRNAs (190 upregulated and 538 downregulated) were identified in PTI versus PT, 126 differentially expressed lncRNAs (72 upregulated and 54 downregulated) were identified in PTI-MMS versus PTI, and 211 differentially expressed lncRNAs (62 upregulated and 149 downregulated) were identified in PT-MMS versus PT. According to the comparison scheme of PaWB-related lncRNAs described in Materials and Methods, 112 lncRNAs were considered as related to phytoplasma infection (Figure 3, Table S7). The 112 phytoplasma-responsive lncRNAs comprised 32 antisense lncRNAs, 1 sense lncRNAs, and 79 intergenic lncRNAs. To confirm their phytoplasma-responsive expression, we selected 17 lncRNAs and validated their expression patterns by qRT-PCR (Table S8, Figure 4). As shown in Figure 4, the qRT-PCR results demonstrated that, except TCONS_00013163, the qRT-PCR results were consistent with those from the RNA-seq data, despite some differences in expression levels.

**Figure 5:**
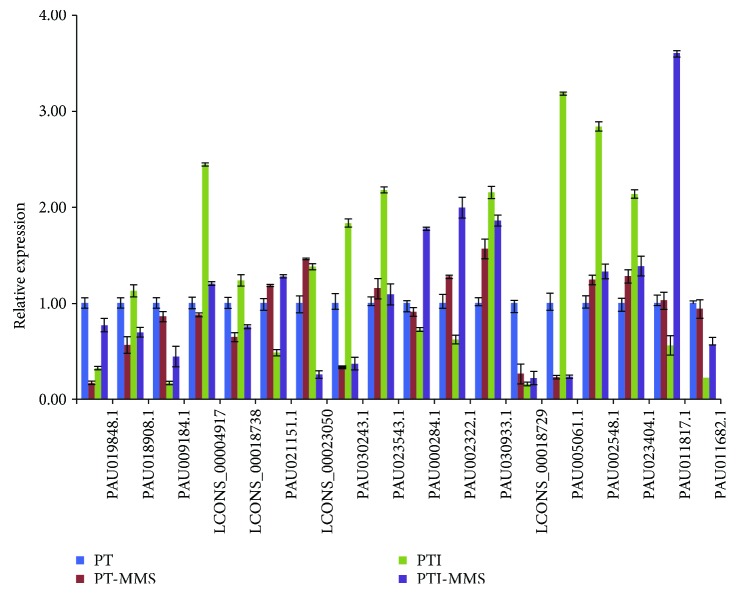
Changes in the relative expression levels of target genes in *P. tomentosa*.

**Figure 6:**
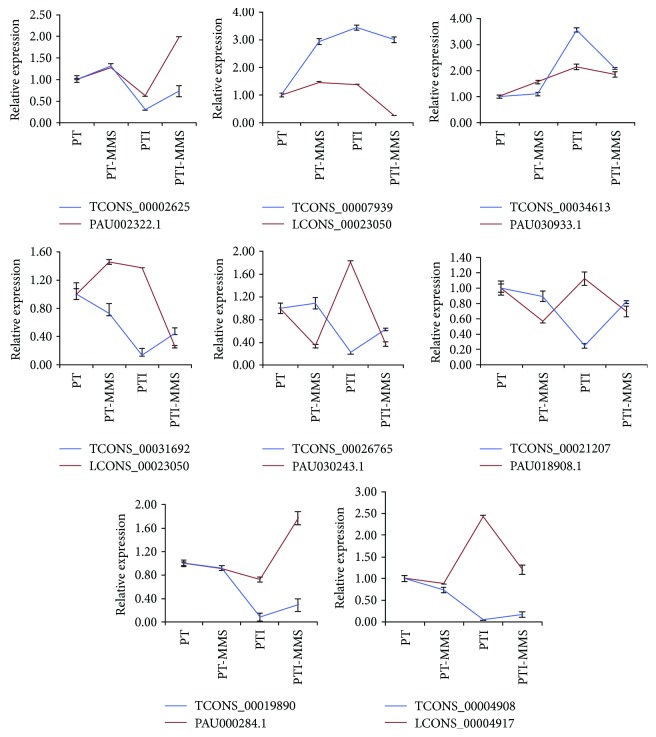
Relative expression of the target genes of eight *P. tomentosa* lncRNAs.

To explore the function of the lncRNAs, we identified and analyzed their target genes. The computational analysis identified 157 potential target genes for 89 lncRNAs. Among them, 86 potential cis-regulated target genes and 71 potential transregulated target genes were predicted for 63 and 72 phytoplasma-responsive lncRNAs, respectively (Table S9). We found that one lncRNA could have more than one target gene, and one target gene could be targeted by one or more lncRNAs. Among these lncRNAs, 33 had one target gene, while two lncRNAs had as many as seven target genes (TCONS_00004908 and TCONS_00004911). Further analysis showed that of the 157 potential target genes, 27 were differentially expressed between the PT and PTI libraries (*P* < 0.05), 14 upregulated and 13 downregulated (Table 3). Moreover, we selected 18 genes and validated their expression by qRT-PCR. The expression patterns of the genes identified by qRT-PCR were consistent with those identified by RNA-seq (Figure 5 and Table S8). Besides, by comparing the expression trends of eight differentially expressed lncRNAs and their target genes, we found that among these differentially expressed lncRNAs-miRNAs pairs, three lncRNA-RNA had a positive correlation (TCONS_00034613/PAU030933.1, TCONS_00002625/PAU002322.1, and TCONS_00019890/PAU000284.1), one lncRNA-mRNA pair (TCONS_00026765/PAU030243.1) had an opposite expression pattern, and the last four lncRNA-RNA pairs (TCONS_00031692/LCONS_00023050, TCONS_00021207/PAU018908.1, TCONS_00007939/LCONS_00023050, and TCONS_00004908/LCONS_00004917) showed the mixed correlation (Figure 6). The same results had also reported in *Populus* [42]. This result suggested that lncRNAs may have various functions in regulating gene expression, and identification and analysis of the relationship between the expression patterns of the phytoplasma-responsive lncRNAs and their potential target genes may help in understanding the functions of these lncRNAs.

To confirm the functional annotations of these 27 target genes, BLAST was used to align their nucleotide sequences to the genes in other plants. The function of 24 of the target genes was confirmed in other plants (Table 3). Among them, eight genes were involved in stress resistance, namely, genes encoding glucan endo-1,3-beta-glucosidase 11 (LCONS_00034335 and PAU030933.1), the acetyltransferase NATA1 (LCONS_00013095), zinc finger CCCH domain-containing protein 9 (LCONS_00004917 and LCONS_00022081), disease-resistance protein (PAU018908.1), protein SRC2 (PAU030243.1), and cytochrome P450 (LCONS_00023050). Seven genes were involved in growth, namely, genes encoding xyloglucan endo-transglycosylase/hydrolase (LCONS_00022082, LCONS_00004912, and PAU019848.1), abscisic acid 8′-hydroxylase (PAU005580.1), zeaxanthin epoxidase (PAU011878.1), MADS-box transcription factor 27 (PAU003690.1), and protein bem46 (PAU021151.1). Four genes were involved in metabolism, namely, genes encoding ribonuclease 3-like protein 1 (LCONS_00019384), ribonuclease H protein (LCONS_00004913), histone-lysine N-methyltransferase (LCONS_00030149), and serine carboxypeptidase II (PAU022614.1). Two genes were involved in transport, namely, genes encoding ATP-binding cassette (PAU011882.1) and calcium-transporting ATPase 12 (PAU023543.1), and two genes were involved in photosynthesis, namely, genes encoding chlorophyll a-b binding protein (PAU002322.1) and photosystem II 10 kDa polypeptide (PAU000284.1). Three of the PaWB-related target genes were annotated as an unknown function, and their functions are still to be verified.

### 3.5. Alternative Splicing Events

**Figure 7:**
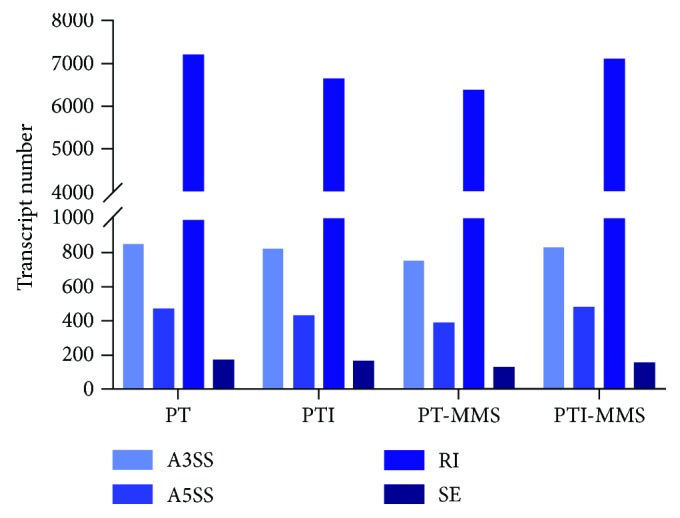
Alternative splicing events in Paulownia. A3SS: alternative 3′ splice site AS events; A5SS: alternative 5′ splice site AS events; RI: intron retention; ES: exon skipping.

Alternative splicing is the key contributor to increasing the diversity of transcripts and proteins encoded in genomes. Many studies have shown that biotic and abiotic stresses can both influence splicing events and that alternative splicing is central for photosynthesis, defense responses, and the circadian clock of plants [66]. Alternative splicing of mRNAs is one of the most reported bioprocesses involving lncRNA; therefore, we calculated the numbers of alternative splicing events based on the Paulownia RNA-seq data and identified four types of alternative splicing: (i) exon skipping; (ii) intron retention; (iii) alternative 5′ splice site; and (iv) alternative 3′ splice site. Among these splicing types, the main patterns of alternative splicing were intron retention, which is consistent with the results of studies in other species [67]. Remarkably, the number of variable splicing in PTI is lower than that of PT, and the frequency of the occurrence of each splicing event is higher than that of PTI (Figure 7). These results indicated that complex variable splicing events had happened and potential differentially expressed proteins had emerged in Paulownia cuttings after phytoplasma infection, which may be due to the defense response of Paulownia triggered by phytoplasma infection. In addition, we found that among the 157 target genes of the PaWB-related lncRNAs, 51 genes were alternatively spliced, resulting in 315 transcripts (Table S10). Further analysis found that these splice variants mainly involved in photosynthesis and carbon metabolism. Genes involved in photosynthesis are known to play significant roles in phytoplasma-infected plants. Previous studies have demonstrated that phytoplasma infection might affect photosynthesis and resulted in yellow leaves. Together, these results suggested that alternative splicing might represent an additional level of gene regulation in response to phytoplasma infection. However, further comprehensive studies of the roles of alternative splicing events in phytoplasma-infected Paulownia are needed.

## 4. Discussion

Understanding the mechanism of gene regulation will provide a molecular basis for PaWB research in Paulownia and contribute to breeding Paulownia that are better adapted to stress conditions. Over the past decade, with the rapid development of sequencing technologies, RNA-seq has allowed the detection of novel types of noncoding transcripts, which has revealed the complexity of eukaryotic genome expression. To date, a large number of lncRNAs have been identified in different species [27–33]. However, phytoplasma invasion activates a set of physiological, biochemical, and molecular responses in host plants, but genome-wide identification and characterization of lncRNAs involved in these responses are poorly studied in Paulownia. In this study, strand-specific RNA-seq was performed to systematically identify and analyze lncRNAs dynamically regulated by phytoplasma infection. Under the strict screen criteria that we used, 3689 high-confidence lncRNAs were identified, of which 112 phytoplasma-responsive lncRNAs comprised 32 antisense lncRNAs, 1 sense lncRNAs, and 79 intergenic lncRNAs. This number of lncRNAs is far less than the numbers of lncRNAs identified in *Arabidopsis* or rice, likely because of the rigorous filtration criteria we used in this study. The structure analysis showed that the 3689 lncRNAs have a median length of 680 bp and usually contain only 1 exon. Our analysis generated a relatively robust list of potential lncRNAs for Paulownia that will be useful for functional genomics research.

### 4.1. Overall Insights into the Conservation of lncRNAs in *P. tomentosa*

Paulownia lncRNAs present low sequence conservation compared to the protein-coding genes, which is consistent with other studies [33, 34, 42]. In our study, most lncRNAs contain one exon and have more specific expression profiles than protein-coding genes. In addition, we found that only limited lncRNAs showed homologues with lncRNAs in other plant species. All these results suggested that lncRNAs identified in this study were not conserved. However, to date, thousands of conserved lncRNAs have been found, possibly owing to the more ancient origins of these lncRNAs giving their functions more time to be stabilized. In fact, the reasons for the limited conservation of lncRNAs are not surprising. First, unlike mRNAs, lncRNAs are not constrained by codon usage and do not have a single long open reading frame to prevent frame-shift mutations. They usually possess short conserved motifs that are not easily identifiable by BLAST and are constrained by structure or sequence-specific interactions [68]. Second, lncRNAs may have undergone recent and rapid adaptive selection. Moreover, some lncRNAs are associated with miRNAs, which can generate from short pairing fragments of lncRNAs that are less constrained in other parts of the transcripts.

### 4.2. Potential Function Roles for Phytoplasma-Responsive lncRNAs

lncRNAs can be targeted either to a nonsense-mediated mRNA decay pathway or to play direct functional roles as transcription regulators. Recent studies indicated that lncRNAs can also act as potential targets of miRNA [43]. In our study, we identified 239 lncRNAs as putative targets of 228 miRNAs. Among them, the pt-miR156q and pt-miR156b were upregulated in phytoplasma-infected cuttings (PTI) and downregulated in MMS-treated phytoplasma-infected cuttings (PTI-MMS). A previous study has reported that miR156 plays a vital role in plant growth and development, for example, in rice overexpressing miR156 showed dramatic morphological changes, including markedly increased number of axillary buds and dwarfism [69]. Similarly, in switchgrass, overexpression of miR156 reduced the apical dominance, delayed the flowering time, caused dwarfism and increased total leaf numbers [70]. Simultaneously, in our study, the lncRNA TCONS_00019806 was downregulated, suggesting that the pt-miR156q might trigger the degradation of lncRNAs TCONS_00019806 and lead to the dwarfism symptom in the phytoplasma-infected cuttings. In addition, miR395 was regulated under sulfate-limited conditions [71], and miR164, miR166, and miR482 have been found to play significant roles in plant microorganism interaction [72, 73]. All these results demonstrated that phytoplasma-responsive lncRNAs may participate in the response to stress.

Target mimicry, a newly identified regulatory mechanism of miRNAs, was first studied in plants and is used to block the interplay between miRNAs and their putative target genes by producing a false target transcript that cannot be cleaved [68]. The effectiveness of lncRNAs that function as putative target mimics for miRNAs has been confirmed in many plants [43]. In this study, we globally analyzed the regulatory network of miRNAs. By bioinformatics analysis, we identified 23 lncRNAs that can act as potential target mimics of 33 miRNAs in Paulownia. Among them, one phytoplasma-responsive lncRNA (TCONS_00021785) was predicted to be the target mimic of the pau-miR319 family. Expression analysis showed that one target gene of the pt-miR319a (PAU012728.1) was upregulated when the expression level of TCONS_00021785 increased after phytoplasma infection, indicating that TCONS_00021785 may regulate the expression of PAU012728.1 (encodes TCP transcription factor) by competing pt-miR319a. Transcription factors control a significant proportion of the defense response by regulating the defense gene. A previous study showed that the expression level of TCPs could regulate by the miR319 [74]. In addition, TCPs can directly determine the expression levels of LOX2, and mutation of TCP binding sites in the *LOX2* promoter strongly reduced its activity [74]. *LOX2* is the key enzyme in jasmonic acid biosynthesis, suggesting that the TCP transcription factor directly controls genes in the JA biosynthesis pathway. This result revealed the potential role of TCONS_00021785 in these processes. However, this hypothesis needs to be further validated, but our results suggest that crosstalk between miRNAs, mRNA, and phytoplasma-responsive lncRNAs may affect many different biological processes and provide useful information for further research into the function of lncRNAs in phytoplasma-infected *P. tomentosa*.

### 4.3. Splice Variants in *P. tomentosa* May Be Related to PaWB

Generation of splice variants is a common mechanism to increase transcriptome plasticity and proteome diversity in eukaryotes. To date, there were few studies that have investigated alternative splicing in response to stress, and no alternative splicing events for genes in phytoplasma-infected Paulownia have been reported yet. In this study, we identified the genes with splice variants that are involved in photosynthesis, plant hormone signal transduction, and carbon metabolism, including genes encoding photosystem II 10 kDa polypeptide (PAU000284.1), phosphoglycerate kinase (PAU000324.1), chlorophyll a-b binding protein (PAU002322.1), abscisic acid 8′-hydroxylase (PAU005580.1), and auxin influx carrier (PAU000910.1). It has been demonstrated that in phytoplasma-infected plants, callose deposition is a common phenomenon and is associated with the accumulation of carbohydrates [75], which can accumulate free hexoses and further repress the synthesis of chlorophyll a-b binding proteins [54]. Chlorophyll a-b binding proteins capture solar energy for the primary light reactions of photosynthesis [76]. The decreased abundance of chlorophyll a-b binding proteins may influence the light-harvesting rate and induce the transfer of electrons [53]. In a previous study, a decreased chlorophyll a-b binding protein has been observed in plants infected with phytoplasma [22, 77]. Besides, photosystem II 10 kDa polypeptide (PsbR) is the main subunit of the oxygen-evolving complex of eukaryotic PSII, which participates in the water-splitting reaction and PSII electron transport [54]. Allahverdiyeva et al. [78] found that lacking of PsbR cloud leads to decrease rates of oxygen evolution and quinone reoxidation. Furthermore, mutation of PsbR in *Arabidopsis* leads to a decreased content of PsbP and PsbQ proteins [79], and plants with lacking PsbP awill be characterized with extensive defects of the thylakoid membrane [80], which is the main place for the transformation of light to the active chemical energy. All these results suggested the significant role of PsbR in PSII system. Moreover, a previous study has demonstrated that phytoplasma infection could lead to a decrease content of PsbR [81], and similar result has also been found in transcriptome and proteome research of phytoplasma-infected Paulownia plant [22, 23]. In this study, the expression of the alternative genes, which encodes photosystem II 10 kDa polypeptide (PAU000284.1) and chlorophyll a-b binding protein (PAU002322.1), was also downregulated (Figure 6), while the lncRNAs TCONS_00002625 and TCONS_00019890 were predicted to regulate the expression levels of the genes that encode chlorophyll a-b binding proteins and photosystem II 10 kDa polypeptide, respectively, implying that after phytoplasma infection, lncRNAs may influence the photosynthesis electron transfer chain in Paulownia.

### 4.4. Phytoplasma Infection Triggers the Immune Responses of Paulownia

Phytoplasma is a plant pathogen that induces drastic malformations, such as short internodes, dwarfism, proliferation of axillary buds, and yellowing leaves. The molecular basis for the pathogenicity of this disease is still poorly understood. Phytoplasma infection altered the expression of genes and proteins in Paulownia [15–23]; however, these observations were descriptive and an in-depth analysis of phytoplasma Paulownia interactions is lacking. Plants infected by phytoplasmas can produce potent strategies to defend themselves against invasion [82, 83]. First, plants perceive the presence of phytoplasma using pathogen-associated molecular patterns (PAMPs) that trigger immunity. In this stage, plants produce a large amount of reactive oxygen species (ROS) and antitoxin, which triggers hypersensitive response. At the same time, phytoplasmas secrete effectors through the Sec secretion system, such as SAP and TENGU, to interfere with the host PAMP-triggered immunity defense signaling transduction and successfully enhance colonization and facilitate their multiplication of themselves in the host plant cells. Plants also utilize cellular receptor proteins to recognize effectors and activate the effector-triggered immunity response, which activates MAPK cascades and induces the disease resistance protein. In the present study, the plant immune responses to phytoplasma infection were activated for we detected genes that encoded enzymes in the signaling pathways (e.g., proline-rich receptor-like protein kinase PERK1) as well as prominent marker genes involved in the associated activities (e.g., disease-resistance protein, glucan endo-1,3-beta-glucosidase, and protein SRC2). In necrotizing viruses-infected tobacco, the glucan endo-1,3-beta-glucosidase increased distinctly [57]. Similarly, in pathogen *Xanthomonas axonopodis* pv. glycines 8 ra-infected peppers, the gene encode for SRC2 was observed highly expressed [60]. Notably, PERK1, disease-resistance protein, glucan endo-1, 3-beta-glucosidase, and protein SRC2 were upregulated in the phytoplasma-infected cuttings (Figure 6). A previous study in Paulownia has also showed that the expression level of gene coding for disease-resistance protein is upregulated after phytoplasma infection [15], while, in this study, lncRNAs TCONS_00021207, TCONS_00034613, and TCONS_00026765 were predicted to regulate the expression levels of genes encoding disease-resistance protein, glucan endo-1, 3-beta-glucosidase, and protein SRC2, respectively. That is to say that these three lncRNAs are likely to play significant roles in phytoplasma-infected cuttings. In addition, the gene encoding cytochrome P450 was also elevated in the PTI cuttings (Figure 6). Cytochrome P450 can serve as an antioxidant to clean up the excess of ROS to reduce the cell damage. Interestingly, in a previous study, the cytochrome P450 level found to be elevated in phytoplasma-infected Paulownia plants [2]. TCONS_00031692 were downregulated and TCONS_00007939 were upregulated in the phytoplasma-infected cuttings, and they both regulated the expression level of gene LCONS_00023050, which encodes cytochrome P450 (Figure 6). Thus, it is clear that in phytoplasma-infected cuttings, lncRNAs might play vital roles in the ROS-induced hypersensitive response and the effector-triggered immunity.

In summary, by using computational analysis, for the first time, we identified 3693 putative Paulownia lncRNAs. These lncRNAs were not conserved among plant species, and some of them were miRNA precursors. Further, we identified 51 target genes of PaWB-related lncRNAs were alternatively spliced, resulting in 315 transcripts. Paulownia lncRNAs perform their function in various ways and their expressions play vital roles in ROS-induced hypersensitive response and the effector-triggered immunity in phytoplasma-infected Paulownia, suggesting the important roles of the lncRNAs in the regulation of biotic stresses. Our analysis also indicated the expression of some lncRNAs could be regulated by miRNAs, but this needs further investigation. The identification and expression analysis of the Paulownia lnRNAs will provide a starting point to understand their functions and regulatory mechanisms in the future.

## Figures and Tables

**Table 1:** Summary of the conserved lncRNAs.

	Total number	Length	Identity	Coverage ≥ 10 number	Coverage ≥ 20 number
*Selaginella*	3	31–38	97–100	0	0
Potato	117	27–644	78–100	31	12
*Vitis*	117	27–335	80–100	38	16
*Zea*	17	26–282	82–100	2	1
*Chlamydomonas*	19	26–65	92–100	1	0
*Physcomitrella*	27	27–73	95–100	2	0
Glycine	36	29–178	80–100	10	4
*Amborella*	38	27–311	80–100	5	4
*Oryza*	59	26–215	79–100	7	2
*Arabidopsis*	36	25–161	82–100	4	1

**Table 2:** lncRNAs acted as precursors of known miRNAs in Paulownia.

lncRNA ID	miRNA ID	Pre-miRNA ID	Pre-miRNA length	Identity	Alignment length	*E* value
TCONS_00000284	pt-miR171d-5p	ssl-MIR171a-p5	90	100	90	2.00*E* − 46
TCONS_00000284	pt-miR171d-3p	mes-MIR171b	90	93	90	2.00*E* − 46
TCONS_00017319	pt-miR160a-5p	ptc-MIR160c	93	95	93	1.00*E* − 36
TCONS_00017319	pt-miR160a-3p	ptc-MIR160c	93	95	93	1.00*E* − 36
TCONS_00017319	pt-miR160c-5p	stu-MIR160a	84	88	84	4.00*E* − 43
TCONS_00017319	pt-miR160c-3p	stu-MIR160a	84	88	84	4.00*E* − 43
TCONS_00019806	pt-miR156e	ptc-MIR156i	100	92	96	2.00*E* − 50
TCONS_00019806	pt-miR156k	gma-MIR156g	142	90	124	4.00*E* − 67
TCONS_00019828	pt-miR156k	gma-MIR156g	142	91	142	1.00*E* − 77
TCONS_00019829	pt-miR156e	ptc-MIR156i	100	92	100	2.00*E* − 52
TCONS_00019829	pt-miR156k	gma-MIR156g	142	100	142	2.00*E* − 77
TCONS_00034513	pt-miR156e	ptc-MIR156i	100	97	91	3.00*E* − 42
TCONS_00034513	pt-miR156k	gma-MIR156g	142	97	92	7.00*E* − 43
TCONS_00034513	pt-miR156q	stu-MIR156c	149	100	149	1.00*E* − 81

**Table 3:** List of identified and characterized PaWB related to proteins in other species.

mRNA ID	PTI/PT	Nr annotation	Function classification	Reference	Species
LCONS_00019384^∗∗^	−1.72	Ribonuclease 3-like protein	Metabolism	Kiyota et al. [48]	*Arabidopsis*
LCONS_00004913^∗∗^	−1.49	Ribonuclease H protein	Metabolism	Cazenave et al. [49]	Wheat
LCONS_00030149^∗∗^	−1.34	Histone-lysine N-methyltransferase	Metabolism	Pavankumar et al. [50]	*Arabidopsis*
PAU022614.1^∗^	−1.57	Serine carboxypeptidase II-3	Metabolism	Bullock et al. [51]	Wheat
LCONS_00018738^∗∗^	1.09	Proline-rich receptor-like protein kinase PERK1	Cell signal transduction	Silva et al. [52]	*Brassica napus*
PAU002322.1^∗^	−1.42	Chlorophyll a-b binding protein of LHCII type 1	Photosynthesis	Chen et al. [53]	*Arabidopsis*
PAU000284.1^∗^	−2.56	Photosystem II 10 kDa polypeptide	Photosynthesis	Allahverdiyeva et al. [54]	*Arabidopsis*
PAU011882.1^∗^	−1.49	ATP-binding cassette, sub-family G (WBC)	Transport	Klein et al. [55]	*Secale cereale*
PAU023543.1^∗^	1.28	Calcium-transporting ATPase	Transport	Boursiac et al. [56]	*Arabidopsis*
LCONS_00034335^∗∗^	2.54	Glucan endo-1,3-beta-glucosidase 11	Stress resistance	Rol et al. [57]	*Sylvestris*; tobacco
PAU030933.1^∗^	2.60	Glucan endo-1,3-beta-glucosidase 11	Stress resistance	Rol et al. [57]	*Sylvestris*; tobacco
LCONS_00013095^∗∗^	2.26	Acetyltransferase NATA1-like	Stress resistance	Lou et al. [58]	*Arabidopsis*
LCONS_00004917^∗∗^	1.37	Zinc finger CCCH domain-containing protein 9	Stress resistance	Maldonado-Bonilla et al. [59]	*Arabidopsis*
LCONS_00022081^∗∗^	14.72	Zinc finger CCCH domain-containing protein 9	Stress resistance	Maldonado-Bonilla et al. [59]	*Arabidopsis*
PAU018908.1^∗^	2.57	Disease-resistance protein	Stress resistance	Fan et al. [15]	*Paulownia*
PAU030243.1^∗^	1.23	Protein SRC2	Stress resistance	Kim et al. [60]	Tobacco; pepper
LCONS_00023050^∗∗^	1.83	Cytochrome P450 71A4	Stress resistance	Liu et al. [2]	*Paulownia*
LCONS_00022082^∗∗^	14.33	Xyloglucan endo-transglycosylase/hydrolase	Growth	Nishikubo et al. [61]	Poplar
LCONS_00004912^∗∗^	−2.28	Xyloglucan endotransglucosylase/hydrolase	Growth	Nishikubo et al. [61]	Poplar
PAU019848.1^∗^	−2.14	Xyloglucan endotransglucosylase/hydrolase	Growth	Nishikubo et al. [61]	Poplar
PAU005580.1^∗^	−2.10	Abscisic acid 8′-hydroxylase 4	Growth	Saito et al. [62]	*Arabidopsis*
PAU003690.1^∗^	5.03	MADS-box transcription factor	Growth	Martel et al. [63]	Tomato
PAU021151.1^∗^	−1.12	Protein bem46-like	Growth	Ramírez et al. [64]	*Pleurotus ostreatus*
PAU011878.1^∗^	−1.09	Zeaxanthin epoxidase	Growth	Audran et al. [65]	*Arabidopsis*
PAU005061.1^∗^	2.03	Uncharacterized protein			
LCONS_00004914^∗∗^	−1.83	Uncharacterized protein			
LCONS_00019769^∗∗^	5.34	Hypothetical protein			

^∗^Represents the known mRNA. ^∗∗^Represents the novel mRNA predicted in this study.

## Supplementary Materials

Supplementary 1 Figure S1: comparison schemes of the four samples.

Supplementary 2 Figure S2: changes of morphology in Paulownia witches' broom cuttings. PT: sample of healthy *P. tomentosa*; PTI: sample of phytoplasma-infected *P. tomentosa*; PT-MMS: sample of 60 mg·L MMS-treated *P. tomentosa*; PTI-MMS: sample of 60 mg·L MMS-treated phytoplasma-infected *P. tomentosa*.

Supplementary 3 Figure S3: detection of phytoplasma 16S rRNA in the four samples. M: marker; 1: phytoplasma-infected *P. tomentosa* cuttings; 2: healthy cuttings of *P. tomentosa*; 3: 60 mg·L MMS-treated healthy *P. tomentosa* cuttings; 4: 60 mg·L MMS-treated phytoplasma-infected *P. tomentosa* cuttings; 5: ddH_2_O.

Supplementary 4 Table S1: primers used for real-time quantitative PCR.

Supplementary 5 Table S2: the number of lncRNAs in each sample.

Supplementary 6 Table S3: conservation of *Paulownia tomentosa* lncRNAs.

Supplementary 7 Table S4: classification of predicted lncRNAs into different noncoding RNA families.

Supplementary 8 Table S5: lncRNAs predicted as a target of miRNAs.

Supplementary 9 Table S6: lncRNAs predicted as target mimicry of miRNAs.

Supplementary 10 Table S7: lncRNAs related to PaWB.

Supplementary 11 Table S8: the FPKM obtained by RNA-seq and the expression obtained by PCR of lncRNA and mRNA.

Supplementary 12 Table S9: the potential target genes of phytoplasma-responsive lncRNAs.

Supplementary 13 Table S10: the splice events of target genes between the healthy and phytoplasma-infected Paulownia.
