# Supplementary figures and images for: Regulation of Long Noncoding RNAs Responsive to Phytoplasma Infection in Paulownia tomentosa
Source: Int J Genomics. 2018 Feb 21;2018:3174352. doi: 10.1155/2018/3174352 (PMC5841072; doi:10.1155/2018/3174352)

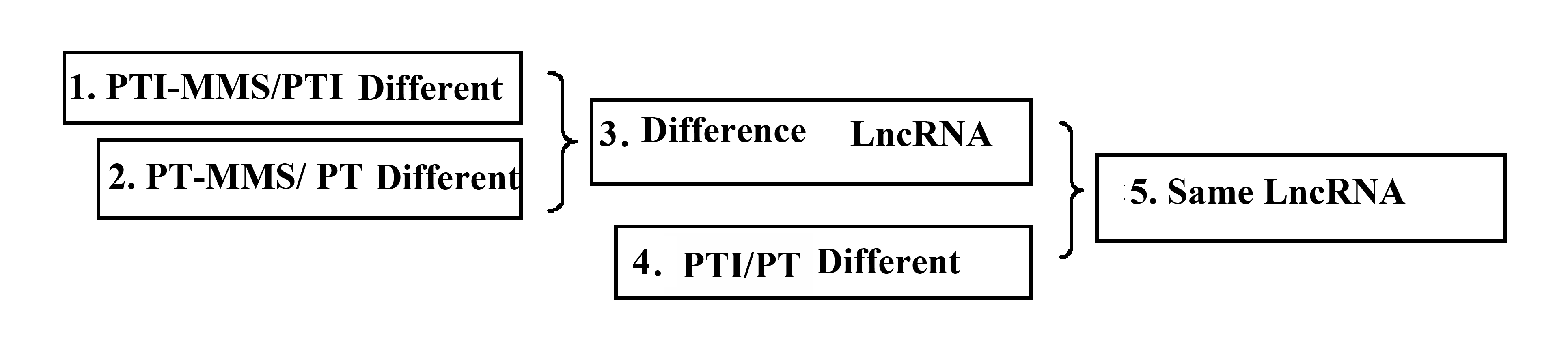

Supplement: Supplementary 1 — Figure S1: comparison schemes of the four samples. [file 3174352.f1.tif]

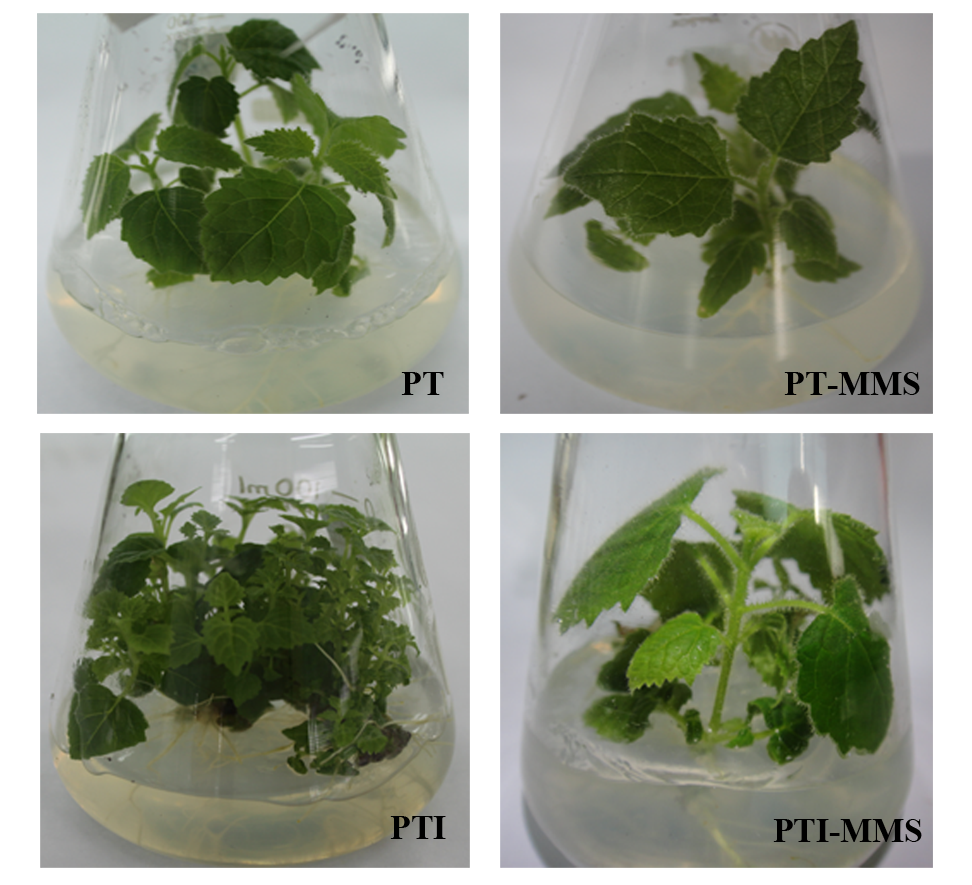

Supplement: Supplementary 2 — Figure S2: changes of morphology in Paulownia witches' broom cuttings. PT: sample of healthy P. tomentosa; PTI: sample of phytoplasma-infected P. tomentosa; PT-MMS: sample of 60 mg·L MMS-treated P. tomentosa; PTI-MMS: sample of 60 mg·L MMS-treated phytoplasma-infected P. tomentosa. [file 3174352.f2.tif]

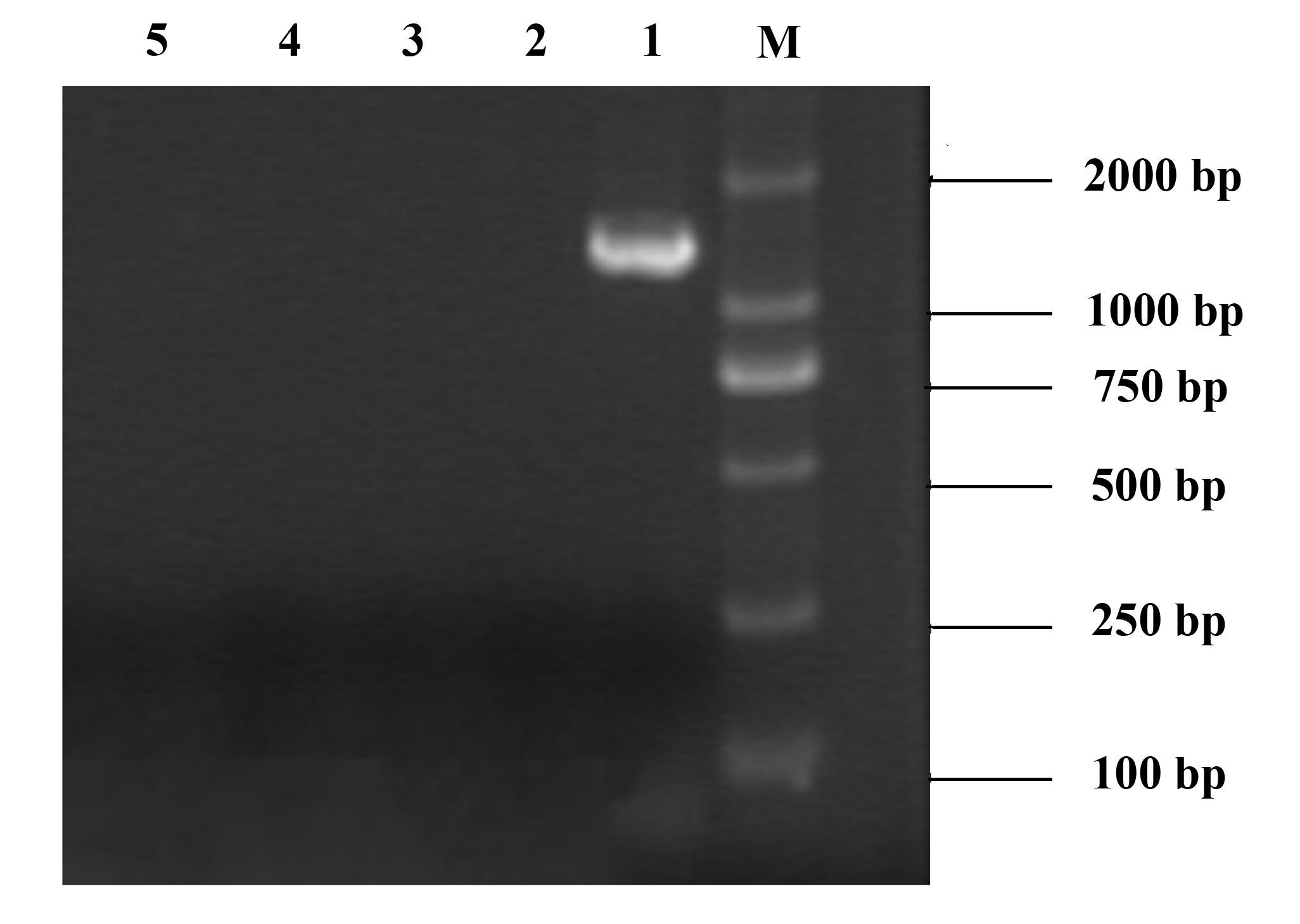

Supplement: Supplementary 3 — Figure S3: detection of phytoplasma 16S rRNA in the four samples. M: marker; 1: phytoplasma-infected P. tomentosa cuttings; 2: healthy cuttings of P. tomentosa; 3: 60 mg·L MMS-treated healthy P. tomentosa cuttings; 4: 60 mg·L MMS-treated phytoplasma-infected P. tomentosa cuttings; 5: ddH2O. [file 3174352.f3.tif]
