# Supplementary material for: Regulation of Long Noncoding RNAs Responsive to Phytoplasma Infection in Paulownia tomentosa
Source: Int J Genomics. 2018 Feb 21;2018:3174352. doi: 10.1155/2018/3174352 (PMC5841072; doi:10.1155/2018/3174352)
Supplement: Supplementary 5 — Table S2: the number of lncRNAs in each sample. [file 3174352.f5.docx]

**Table S2 The number of lncRNAs in each sample**

|  | PT | PTI | PT-MMS | PTI-MMS |
| --- | --- | --- | --- | --- |
| Number | 3414 | 3504 | 3371 | 3521 |
